# Supplementary material for: A Novel Double Mosaic Virus-like Particle-Based Vaccine against SARS-CoV-2 Incorporates Both Receptor Binding Motif (RBM) and Fusion Domain
Source: Vaccines (Basel). 2021 Nov 5;9(11):1287. doi: 10.3390/vaccines9111287 (PMC8619050; doi:10.3390/vaccines9111287)
Supplement: Supplementary file 1 [file vaccines-09-01287-s001.zip › vaccines-1426458-supplementary.pdf]

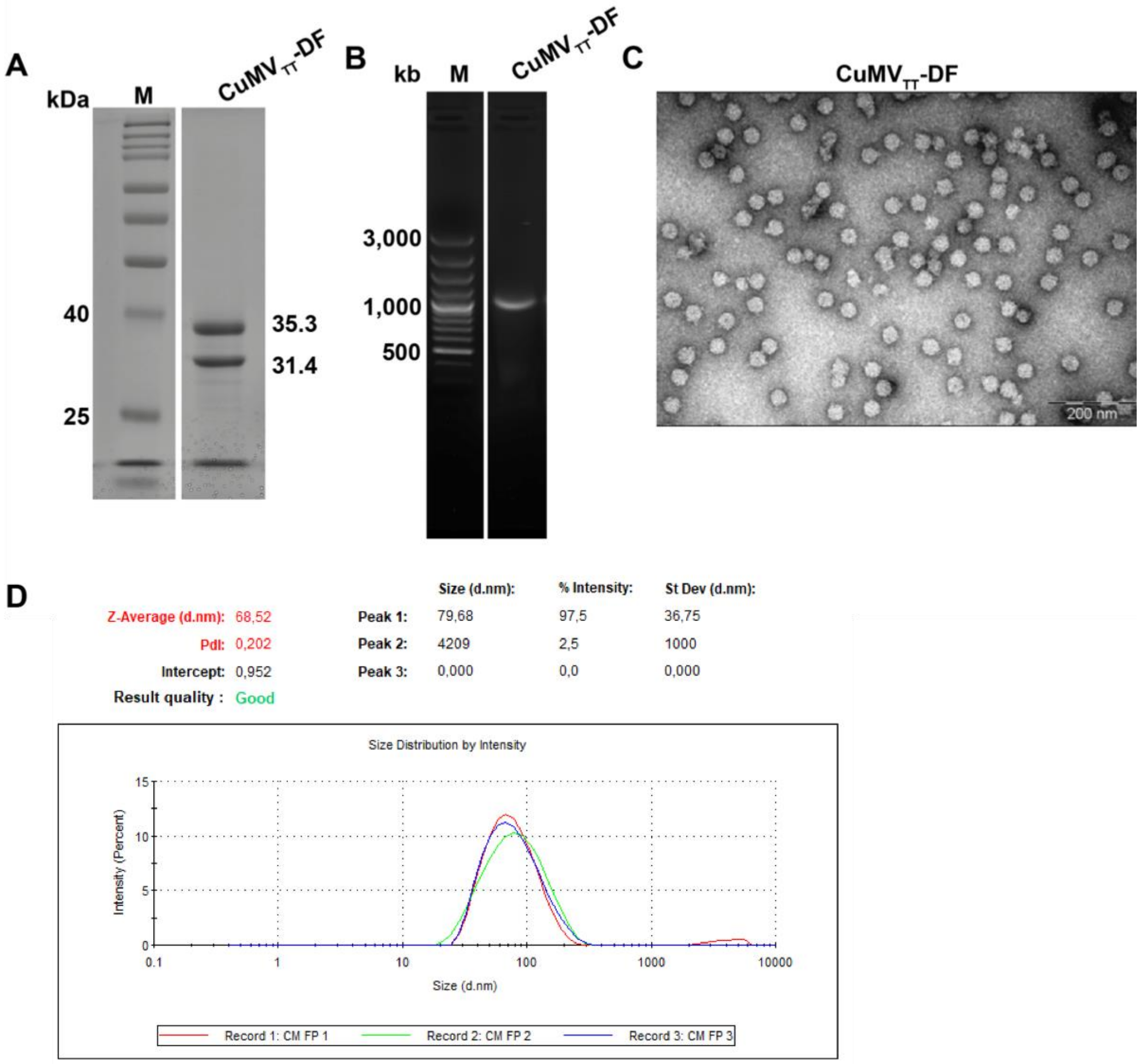

Figure S1. SDS-PAGE (**A**) and agarose (**B**) gel analysis of CuMV$_{TT}$-DF vaccine after storage at 4 °C for 6 months; TEM (**C**) and DLS (**D**) analysis of CuMV$_{TT}$-DF vaccine after storage at 4 °C for 12 months.
